# Do Humans Really Prefer Semi-open Natural Landscapes? A Cross-Cultural Reappraisal

**DOI:** 10.3389/fpsyg.2018.00822

**Published:** 2018-05-29

**Authors:** Caroline M. Hägerhäll, Åsa Ode Sang, Jan-Eric Englund, Felix Ahlner, Konrad Rybka, Juliette Huber, Niclas Burenhult

**Affiliations:** ^1^Department of Work Science, Business Economics and Environmental Psychology, Swedish University of Agricultural Sciences, Alnarp, Sweden; ^2^Department of Landscape Architecture, Planning and Management, Swedish University of Agricultural Sciences, Alnarp, Sweden; ^3^Department of Biosystems and Technology, Swedish University of Agricultural Sciences, Alnarp, Sweden; ^4^Centre for Languages and Literature, Lund University, Lund, Sweden; ^5^Department of Linguistics, University of California, Berkeley, Berkeley, CA, United States; ^6^Institut fûr Information und Medien, Sprache und Kultur, University of Regensburg, Regensburg, Germany

**Keywords:** landscape preference, consensus, Western sampling bias, experts/novices, cultural and linguistic diversity

## Abstract

There is an assumption in current landscape preference theory of universal consensus in human preferences for moderate to high openness in a natural landscape. This premise is largely based on empirical studies of urban Western populations. Here we examine for the first time landscape preference across a number of geographically, ecologically and culturally diverse indigenous populations. Included in the study were two urban Western samples of university students (from southern Sweden) and five non-Western, indigenous and primarily rural communities: Jahai (Malay Peninsula), Lokono (Suriname), Makalero (Timor), Makasae (Timor), and Wayuu (Colombia). Preference judgements were obtained using pairwise forced choice assessments of digital visualizations of a natural landscape varied systematically on three different levels of topography and vegetation density. The results show differences between the Western and non-Western samples, with interaction effects between topography and vegetation being present for the two Swedish student samples but not for the other five samples. The theoretical claim of human preferences for half-open landscapes was only significantly confirmed for the student sample comprising landscape architects. The five non Western indigenous groups all preferred the highest level of vegetation density. Results show there are internal similarities between the two Western samples on the one hand, and between the five non-Western samples on the other. To some extent this supports the idea of consensus in preference, not universally but within those categories respectively.

## Introduction

Understanding human preferences for landscape, and particularly to what degree preferences differ between populations is of importance both from a basic research perspective and from a practical landscape and environmental management perspective. A high consensus in preference across populations would enable general theoretical models of landscape appraisal and justify the application of general models of landscape management across cultures.

Since the mid-1970s, several well-established and oft-cited paradigms in environmental psychology have advocated a universal consensus in human preferences for certain types of natural environments. Such paradigms include the Prospect-Refuge theory (Appleton, 1975), the Savannah Theory (Orians, 1980), the Biophilia Theory (Fromm, 1964; Wilson, 1984; Ulrich, 1993), and The Preference Matrix (Kaplan and Kaplan, 1978, 1982, 1989). At the heart of these theories lies the idea that human adaptions during evolution have led to the development of innate preferences for particular environments; landscapes with physical characteristics that support psychological dimensions like understanding and exploring the environment, and feeling safe in it. Many of these theories make a clear connection between preference and visibility, stating for example that high preference will occur for landscapes where you can see without being seen (Appleton, 1975), landscapes with open expanses with clusters of trees (Orians, 1980), or landscapes which are visually understandable and offer possibilities for exploration (Kaplan and Kaplan, 1989). These hypotheses are one reason why landscape preference research is dominated by work on the visual modality but this practice is further enforced by the convenience of testing with visual stimuli, as compared to the provision of multi-sensoric stimuli which demands a more complex experimental setup.

The vast majority of empirical landscape preference studies also suffer from a sampling bias toward urban Western populations. Although a growing number of studies target the general public or particular stakeholder groups such as residents or recreationists (Soliva et al., 2010; Ode Sang and Tveit, 2013; Junge et al., 2015), university students are overrepresented as sample populations. Some examples of work using students as respondents in this area of work are the research by Strumse on visual preference for agrarian landscapes in Norway (Strumse, 1994, 1996), studies by Herzog (Herzog, 1984, 1989) and others conducted in the context of Kaplan and Kaplan's Landscape Preference Matrix, and many recent studies in China and other Asian countries (Zhao et al., 2013a; Wang et al., 2016). There is a growing awareness within the behavioral sciences that such populations are not necessarily the most representative ones for generalizing about humans. Indeed, they form outliers in a range of fundamental psychological and behavioral domains (Henrich et al., 2010)^1^. At least in the context of the more general question of pan-human consensus in preference for particular gross properties of landscapes, the inclusion of much more diverse samples of respondents will be necessary in order to reach reliable conclusions.

The degree of visual openness of the landscape is central to some of the key concepts in the paradigms of environmental psychology cited above (Tveit et al., 2006). In some form or other, degree of openness has consequently been a frequent variable in the empirical studies of landscape preference, and, generally, moderate to high openness has been preferred, at least by the North American, European and East Asian sample populations on which such studies have been typically carried out (Ulrich, 1993). Studies also draw attention to the fact that, in cultures where environments are manipulated by humans for aesthetic pleasure, i.e., parks and gardens, such environments are often half-open landscapes with water features and resemble a savannah-type landscape (Orians, 1980, 1986). Two empirical photo-based studies of different biomes give some support to the idea of an innate preference for savannah over tropical rainforest, desert, temperate deciduous forest and coniferous forest (Balling and Falk, 1982; Falk and Balling, 2010). The results suggest this preference for savannah is present in childhood but then declines with age and experience (as a result of familiarity and enculturation). A third similar study of different biomes (desert, tundra, grassland, coniferous forest, deciduous forest and tropical forest) found tundra and coniferous forest to be the most favored biomes, and hence not supporting the Savannah Theory (Han, 2007). However, theories and studies like those above consistently investigate general preferences for a landscape scene *as a whole*. Openness is then implicitly recognized as one structural component making up the scene. However, the significance of openness is likely to be a more complex issue when studied in a specific context where openness might have a particular functional importance or be commonly associated with the type of landscape. For instance, in a study focusing on visual scale of agricultural landscapes in Norway, Tveit (2009) found that both degree of open land in the landscape as well as size of landscape rooms were predictors of preference for students in landscape professions but not for the general public. This indicates that expertise or special interests as well as the typology of landscape might influence the preferred degree of openness.

On a basic structural level landscape openness is a product of topography and vegetation, where vegetation in itself plays an important role in the landscape preference studies as its presence or absence often defines what is labeled natural or urban built or human-influenced landscapes in the study design. In comparisons of general preferences for natural vs. built environments studies have consistently found natural environments to be preferred over built ones (Kaplan and Kaplan, 1989). It could be argued, however, that both the context of comparing natural to built environments and the limited range of settings studied are major shortcomings. Studies of variations within a built or a natural scene type are rarer and the majority of studies are focused on everyday environments in urban settings, limiting the available data for more wild or pristine natural environments. To people in industrialized societies the category of “nature” includes not only wilderness but also for instance agricultural land and golf courses (Ulrich, 1993). Studies have shown that European, North American and Japanese respondents tend to think of the environment as natural if it is dominated by features like vegetation, mountains and water as opposed to environments that contain buildings and urban objects like cars and signs (Ulrich, 1993).

The basic research interest in understanding human responses to landscape is only one of the incentives for landscape preference research. Equally important is the need for general models and systematic assessments of human experiences of landscape in environmental management (Daniel, 2001). In planning and management of environments it is important to take into account the opinions and needs of various stakeholders. High consensus and generalizability of results would of course be desirable in practice. Although there is a long-standing debate about the consensus assumption (Van Den Berg et al., 1998) and its evolutionary basis (Joye and Van Den Berg, 2011), not many empirical studies have made a serious attempt at investigating it on the more cross-cultural level.

There are only very few studies that include respondents from non-Western and non-urbanized populations as well as from more non-Western landscape settings. Rare exceptions of photograph-based quantitative preference studies in non-Western contexts are the studies by Falk and Balling (2010) and Sonnenfeld (1967). With the explicit aim of extending their previous research on innate preferences for savannah, mostly based on Western populations, to a different cultural and environmental context, Falk and Balling (2010) used three different samples from River State, Nigeria. One of the samples consisted of students from a technical college who lived spread out in the area of River State (delta and upland area). The other two samples were secondary school children from an area with mature tropical rainforest and from an area with mangrove forest. The majority of the respondents in these two samples had no experience of landscape types other than the one they lived in. As in the previous study (Balling and Falk, 1982), the results showed that savannah was preferred as the place to live over tropical rainforest, desert, temperate deciduous forest, and coniferous forest.

In the study by Sonnenfeld (1967) students and teachers of Inuit populations in three villages in Alaska were compared with various non-indigenous professional groups living and working in the Arctic as well as a sample of students in Delaware. The study revealed differences both between and within indigenous Arctic and non-indigenous Arctic populations. Non-indigenous had higher preference than natives for rugged landscapes and more vegetation. Furthermore, the Delaware students ranked significantly higher on vegetation than the other non-indigenous groups working in the Arctic, but lower on topography. As expected there was also variation between the indigenous Inuit groups, where for instance the inland caribou hunting group ranked highest on topography and vegetation in comparison to the other two Inuit groups who were from villages on the coast. Quantitative studies with indigenous populations like the one by Sonnenfeld are extremely rare. Landscape preference and perception of indigenous populations is to some extent studied within natural resource management, where there has been a particular concern with how landscape changes through management practices for production and conservation have an impact on indigenous people living in affected areas (Lewis and Sheppard, 2005, 2006; Lewis, 2008, 2010). These are however typically context-dependent case studies aimed at understanding the perceptions of a specific area by the local inhabitants.

As the above overview makes clear, there is a lack of quantitative studies of how preferences of general landscape structure and content might be similar or different across a broad sample of geographically, culturally and linguistically diverse populations. The study presented in this paper was designed to test the assumption of universal consensus in preferences for natural landscapes, and particularly for moderate to high openness in the landscape, by including culturally diverse populations from a wide range of geographical settings. Our sample populations are represented by small-scale and lesser-known indigenous communities with a close and largely traditional relationship to their environment. The sample also includes two university student populations (one of which is involved in landscape studies and one which is not) in order for us to compare how more conventional sample populations respond to the stimulus across experts and non-experts. In addition to this sampling of an unprecedented cross-section of humanity, our approach breaks new ground in that it represents an interdisciplinary collaboration between landscape studies and field linguistics. Thus, language experts with established field sites provide practical and communicative access to the diverse sample and enable a culturally and linguistically attuned experimental protocol in each of our sample populations.

## Methods

We developed an *in-situ* choice experiment for landscape preference using computer generated visualizations. Within landscape preference research the use of ranking or rating of images on a Likert scale has been the dominant method for establishing preference (Kaplan and Kaplan, 1989). However, it has been suggested that choice experiments more closely correspond to real world behavior and are therefore more suitable for analysing landscape preference (Arnberger and Eder, 2011). The usefulness of choice experiments is likely to be greater when comparing landscapes with large similarities since it forces a ranking between landscapes, which the Likert scale does not. While choice experiments are still rather rare within landscape preference research, they have been more frequently used to gain a monetary evaluation of different type of policies and management strategies related to landscape preference (e.g., Nielsen et al., 2007; Dachary-Bernard and Rambonilaza, 2012; Grammatikopoulou et al., 2012).

### Visual stimuli

The present study uses state-of-the-art digital visualizations of natural landscape. In this study there are several advantages for choosing this type of visual stimuli, rather than photographs of real natural landscapes. Visualizations provide imagery that is constant across scenes when it comes to general parameters like weather, lighting, vegetation type, and ground texture. Visualizations are also superior to photographs for systematically varying the parameters to be tested, in this case topography and vegetation density. Apart from offering absolute control over the test parameters, visualizations also minimize the risk of scene content being familiar or having a cultural significance to a particular group. Focussing on assessment of landscape openness, we considered it an advantage to use imagery that is recognizable as a landscape but neutral in relation to the wide variety of landscapes and cultural groups that the field sites represent. Concerns might be raised that respondents could be unfamiliar with computer generated imagery. However, visualizations have been shown in several studies to be comprehensible and a valid substitute for photographs for making assessments (Daniel, 2001; Meitner et al., 2005; Pihel et al., 2014). This has also been shown to be the case for people with limited experience with visualizations (Lewis and Sheppard, 2006).

**Figure 1:**
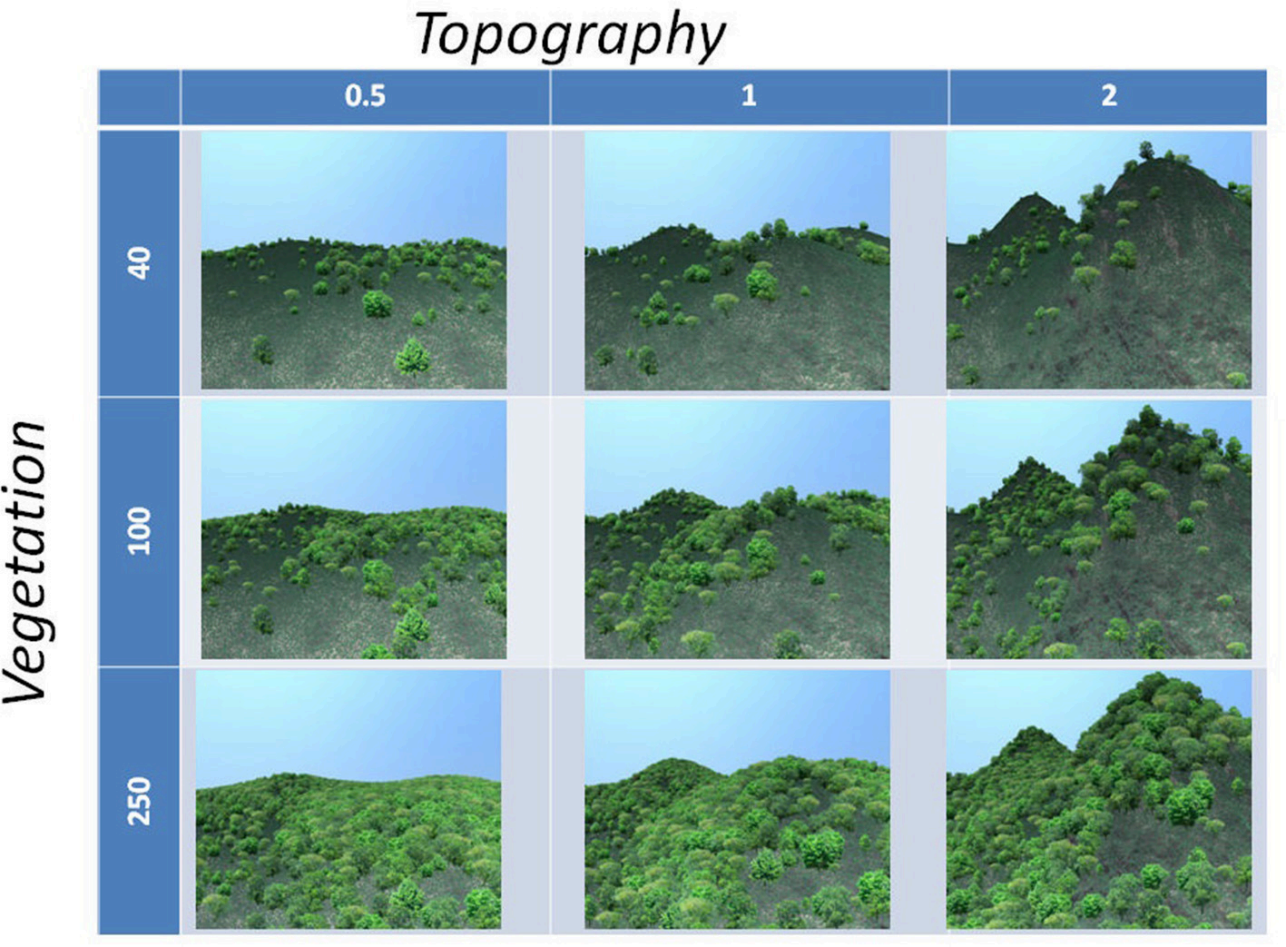
Visual stimuli. The nine images used in the study, with rows showing amplitude of elevation variation and columns showing increased density of vegetation.

The imagery for the study was optimized to study respondents' reactions to two major structural components affecting the openness of the landscape: topography and vegetation density. The two components were varied systematically on three different levels (high, medium and low) resulting in 9 different scenarios for the same base landscape. In order to develop images of these scenarios, a neutral landscape was created in the form of a virtual model using the software Virtual Nature Studio, based on the methodology presented in Ode Sang et al. (2014). The neutral landscape used an elevation map developed in ArcGIS and a standardized ecosystem with regards to tree and bush species present as well as ground texture. The distribution of vegetation across the terrain was also standardized. The nine different scenarios were created by varying the amplitude of elevation and density of the vegetation. Within this virtual landscape a fixed view point was selected and one image for each scenario was rendered using a fixed direction and angle, resulting in the nine images shown in Figure 1.

### Sample populations

Our study includes five non-Western, indigenous and primarily rural communities: Jahai (Malay Peninsula), Lokono (Suriname), Makalero (Timor), Makasae (Timor), and Wayuu (Colombia), whose landscape preference has not previously been investigated. We also included two Western (Swedish) university student samples, i.e., common samples on which much landscape preference work is based. There are conflicting results on whether experts and non-experts differ or agree on landscape preferences. However, in some studies where university students from landscape architecture and landscape oriented fields were used, results showed that they differed in preference from students of other disciplines (Buhyoff et al., 1978; Herzog et al., 2000) as well as from the general public (Tveit, 2009). For this reason our study included both university students from the humanities and university students of landscape architecture.

The sample populations are diverse in terms of their geographical distribution (Europe, Mainland Southeast Asia, Australasia, and South America), linguistic affiliation (the Arawakan, Austroasiatic, Indo-European and Timor-Alor-Pantar language families) and primary subsistence mode (foraging, slash-and-burn agriculture, and industrialized). Habitats vary topographically and vegetationally, but all populations except the Swedish ones are found in tropical regions. However, the samples also offers opportunities for comparison of closely connected populations speaking related languages and inhabiting similar environments: the Lokono and the Wayuu, the Makalero and the Makasae, and the two Swedish groups.

The study included a total of 140 respondents, and the sample size for each of the seven groups varied between 15 and 25 participants (for sample details see Table 1). The availability of participants varied considerably between the field sites. The non-Western samples are small and endangered communities to start with. Hence it is very difficult to achieve samples which are perfectly matched for common background factors like age and gender. Furthermore, the diverse cultural contexts and different habitats and residence influence and limit the possibilities for balanced sampling on factors that could have been interesting to analyse and control for in a cross cultural study, such as rural/urban residence or education level. The aim and originality of this study is its interdisciplinary approach that enables the inclusion of indigenous populations so far not studied, and the comparison of such populations with conventional Western student samples. This allows us to provide a first insight into landscape preferences across very diverse groups. To illustrate the diversity contained in our sample, the following sections describe the cultural, linguistic and environmental specifics of each population.

**Table 1:** The cross-cultural landscape preference sample.

**Ethnolinguistic community**	**Country**	**No. of participants**	**Mean age**	**Experiment setting**	**Contact language used by experimenter**	**Instruction (transcribed, glossed, translated)**	**Collector**
Jahai	Malaysia	17 (Females: 2, Males: 15)	36 (18–65)	Indoors	Jahai	Mεy tmpət k = tmpət ns-gɔs btʔεt btol? What place REL = place NMZ-to.dwell to.be.good SUPER “Which place is the best place to dwell in?”	Burenhult
Lokono	Suriname	25 (Females: 13, Males: 12)	67 (41–87)	Outdoors	Lokono	Halo-n kaku-ti = da = bo where-LOC live-DESI-IMED = 2Osg “Where do you want to live?”	Rybka
Makalero	East Timor	19 (Females: 4, Males: 15)	42 (23–76)	Mostly outdoors	Makalero	Fi so'ot = ini muʔa taure-isi-nomar? 1pi want = CONJ land which:REDUC-be.at:REDUC-dwell:BOUND “On which land do you want to dwell?”	Huber
Makasae	East Timor	15 (Females: 4, Males: 11)	25 (15–59)	Mostly indoors	Makasae	Fi karak muʔa waʔa nahigalu isi ni-oma gini? 1pi want land REL where be.at REFL-house make “On which land do you want to build your house?”	Huber
Swedish 1, Swedish University of Agricultural Sciences, SLU	Sweden	21 (Females: 18, Males: 3)	25 (23–40)	Indoors	Swedish	Välj den bild som visar var du helst skulle vilja bo Choose that picture REL show-PRES where 2sg rather would to.want to.dwell “Choose the picture that shows where you would rather dwell”	Hägerhäll, Sang
Swedish 2, Lund University	Sweden	23 (Females: 17, Males: 6)	24 (19–42)	Indoors	Swedish	Välj den bild som visar var du helst skulle vilja bo choose that picture REL show-PRES where 2sg rather would to.want to.dwell “Choose the picture that shows where you would rather dwell”	Ahlner
Wayuu	Colombia	20 (Females:10, Males: 10)	41 (27–63)	Indoors	Spanish	¿ dónde preferirí-as vivir? where prefer-2sg live? “Where do you prefer to live?”	Rybka

#### Swedish (Sweden)

Sweden is a highly urbanized country with 85% of its population living in towns and larger settlements (United Nations, 2014). The two universities from which students were recruited are situated in southernmost Sweden. Most of the students were from the southern part of Sweden (the Götaland and Svealand regions), which encompasses several different landscape types, which however share some commonalities. The southernmost area (where the two universities are located) is dominated by flat agricultural land, as are the areas around the big lakes further north. Other areas of Götaland and Svealand are dominated by densely forested plateaus with industrialized forest production.

The landscape architect students participating in the study were based at the Swedish University of Agricultural Sciences, SLU, in Alnarp in the south of Sweden. All the students recruited speak Swedish as their first language. The students were all in the fourth or fifth year of studies and have been exposed to environmental psychology theories as well as studies of the natural and cultural processes of landscape through their training. They have lived in this part of Sweden for the period of their study. The participants from Lund University were mainly undergraduate students of linguistics and speech therapy, whose first language was Swedish. These students had not taken part in any university courses related to landscape or environmental studies.

#### Jahai (Malaysia)

The Jahai are mobile subsistence foragers in the Malay Peninsula, Southeast Asia. They number c. 1,000 and speak a language belonging to the Austroasiatic language family. Their territory comprises a landlocked area topographically dominated by the Titiwangsa Range. The relief ranges between 100 and 1,800 m, and the area forms a maze of narrow, steep-sided valleys drained by countless streams and rivulets. Primary Dipterocarp rainforest forms a dense cover over most of the territory. Jahai existence is firmly associated with the mountain rainforest: they move and dwell within it; they subsist on its wild resources; their belief system is structured by it; and their linguistic and cognitive categorization strategies reflect deep knowledge of it (Burenhult, 2008). Like other, related forager groups in the peninsula (but unlike their sedentary agricultural neighbors), the Jahai prefer the forest environment to tree-cleared land because it is considered “cool” and therefore “healthy” (Benjamin, 1985). Nowadays most Jahai are resettled in government-sponsored villages. Many younger individuals have received basic schooling in the unrelated majority language Malay.

#### Makalero (East Timor)

Makalero is an ethnolinguistic group of some 7,000 in the Iliomar subdistrict of East Timor's easternmost district, Lautém. The subdistrict occupies a 30 km long section of the south coast and stretches some 10 km inland up into the central mountain range. The landscape is mountainous, with elevations ranging from sea level to almost 900 m, and mostly covered with tropical dry and moist deciduous forest. Agricultural land is scattered through the forested areas. Several rivers cross the country, draining into the Timor sea; only two carry water year-round. The larger of these, the Irabere river, forms the western boundary of the Makalero-speaking region. The subdistrict's coastline is characterized by relatively rough seas. Makalero speakers are mainly clustered in and around Iliomar town, some 5 km distant from the coast. The subdistrict's population are predominantly small-scale farmers practizing shifting cultivation; the sea plays a very minor role in their life and subsistence. The main crops cultivated are rice, maize and vegetables. The majority of the population identify as Catholics; even so, animist traditions remain important in daily life. Landscape features such as mountain tops and water bodies play an important role in this tradition. In particular, mountain tops and water bodies that are associated with clan history are considered sacred and access to them is forbidden to uninitiated people.

#### Makasae (East Timor)

The Makasae, a group very closely related linguistically to the Makalero, comprise some 90,000 people over a large area in eastern East Timor. The data for this study were collected in Baucau, the nation's second-largest urban center with a population of about 20,000.

Baucau is situated on the island's north coast. The colonial-era old town is built into a carstic cliff face at an altitude of 300–400 m. There is a steep slope, approximately 2 km in length, down to the coast. Numerous small watercourses have their source in the area. The new town was built during the Indonesian occupation (1975–1999) on the plateau above the old town, at an altitude of approximately 500 m. It is separated from the old town by a distinct rock escarpment and is rather arid. Unemployment is high, and small-scale farming, fishing, as well as small livestock form an important part of the inhabitants' subsistence. Like the Makalero, the Makasae are Catholic, but retain animist traditions in which parts of the landscape associated with clan history are considered sacred.

#### Wayuu (Colombia/Venezuela)

The Wayuu inhabit the Guajira Peninsula, a border area between Colombia and Venezuela on the Caribbean coast. The territory is dominated by arid, semi-desertic landscapes with scarce xerophytic vegetation and hardly any permanent water features. Topographically, the northern and central parts of the peninsula are mostly flat (maximally 865 m) but the terrain rises in the south, toward the Sierra Neveda de Santa Marta (5,700 m) and Serranía del Perijá (3,630 m) ranges. This part of La Guajira is also more humid and occasional wetlands appear. The majority of the Wayuu practice goat herding and live in small settlements called *rancherias*, usually quite distanced from one another to prevent herd mixing. The Wayuu adopted this pastoralist lifestyle shortly after contact with the Westerners. Though the Wayuu are quite a numerous indigenous group (around 450,000) with a thriving culture, their language (which belongs to the Arawakan language family) is today endangered, as it is not being transmitted to children.

#### Lokono (The Guianas)

The Lokono people inhabit the northern parts of French Guiana, Suriname and Guyana. The landscape of the area where the data were collected is characterized by higher lying savannahs dissected by gently sloping creek valleys, whose vegetation consists mostly of dense tropical rainforest. The many local watercourses are characterized by tidal and seasonal differences in water height and flow, frequent meanders and anastomosing channels. The differences in elevation rarely exceed 50 m. The Lokono villages are located on the edge of the forest and the savannah, on cleared areas called *mawkili* “lit. empty area” or *sawkili* “good area.” Today, many Lokono have partly given up the traditional slash-and-burn agriculture and adapted to the cash economy. Hunting, fishing and gathering have lost their status as subsistence practices. With most villages being reachable by road, traditional transport by water has disappeared in most places. The Lokono language (Arawakan language family, closely related to Wayuu language) is spoken today only by a few elders in each village.

### Assessment of preference and familiarity

The phrasing of the preference question was given special consideration in this study, due to the linguistic and cultural diversity of the sample populations, as well as the practical aspects of administering the task in the field settings. Rather than asking for a general preference, which leaves undesirably large room for interpretation, we decided to put preference in a context that would be more expressible and understandable across field sites. Thus we chose to focus our question on habitation preference. This approach was also used in relevant previous studies (Sonnenfeld, 1967; Balling and Falk, 1982; Falk and Balling, 2010), where it was intended to direct respondents away from judging the purely aesthetic visual aspects of the image and thus provide a context more relevant to the theoretical claims (Balling and Falk, 1982). It has also been suggested that habitation preferences would be more cognitively based and hence more appropriate for detecting differences between groups with varying experience and education (Ulrich, 1993). Differences between settings are said to become more significant when they are judged as places to live in rather than places to see or visit (Balling and Falk, 1982).

A further consideration concerned the translatability and cultural appropriateness of the preference question. The English phrasing “Where would you prefer to live?” presents several potential translational difficulties. The most apparent one is how to understand and convey the indigenous notion of habitation. For example, the English term *live* conflates life and habitation and can mean both “to be alive” and “to dwell,” whereas Swedish (in our sample and closely related to English) makes a lexical distinction between the two: *leva* “to be alive, to exist” and *bo* “to dwell, to have one's home.” Translation into some other languages requires a paraphrase like “to build one's house,” in order to specifically express habitation (e.g., Makasae, in our sample).

An additional challenge here is the notion of permanence. For example, English *live* encodes permanent habitation (unlike *stay*, as in a hotel), whereas Swedish *bo* is unspecified as to permanence. In our sample, permanence also has a cultural twist: one population is traditionally nomadic (Jahai) and until recently erected only temporary camps, intended for habitation for a few months, at the most. Inevitably, any notion of permanence in such a setting will be appraised differently from one in which more sedentary dwellings are the norm. Drawing on our linguistic expertise from the sample settings and taking the indigenous notions of habitation into full account, we have aimed to design the linguistically and culturally most attuned preference question for each setting. The questions are presented orthographically, glossed and translated in Table 1.

The study also includes an assessment of the respondents' familiarity with the presented landscapes, operationalized by respondents indicating which single image in the whole set was most similar to the landscape in which they grew up. This is further explained in the procedure section.

### Procedure

#### Survey

Participants at both Swedish universities were recruited by the researcher, who visited classes and gave information about the study and instructions on how to sign up. Participation was voluntary and gave no course credits but a cinema ticket was offered to all who participated. Among the other populations volunteers were recruited following oral presentation of the study at informal community gatherings organized for the purpose by the respective researcher together with community representatives. The researchers have long-term experience of working in the respective communities. The research was explained as a picture task involving pictures of the land. Participants were offered a small incentive according to locally established practices. Sessions typically lasted 5–10 min.

**Figure 2:**
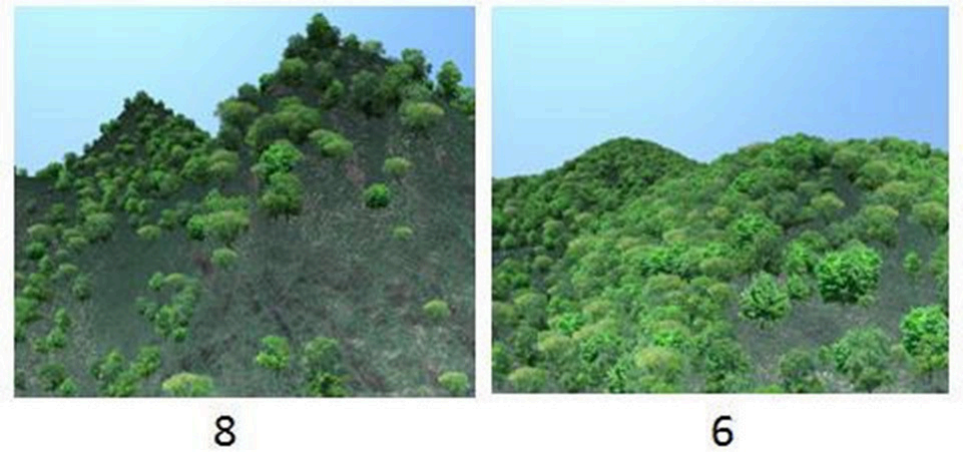
Example of image pair as it appeared to the respondents in the choice situation.

Each of the nine images was paired with the others, resulting in 36 unique image pairs. For an example, see Figure 2. The image pairs were shown to the respondent as printed and laminated A4 sheets, with one image pair on every A4, with the image size 14 × 12 cm.

Respondents were tested individually with a researcher present the whole time. Swedish respondents performed the study indoors while for the other sites the environments in which the test was done varied due to practical conditions, see Table 1. For each image pair the respondent was asked to choose in which of the two environments they would prefer to live. In all but one sample setting respondents were instructed in their respective native language; the one exception was the Wayuu, who were instructed in Spanish—the second or sometimes first language of the Wayuu people today. The question was tailored by each language expert to express the target meaning of habitation preference (see section Visual Stimuli and Table 1). For practical reasons the same presentation order of the image pairs was used for all respondents and all sites. This order was created through randomization to avoid bias. After the 36 pairs had been shown, each respondent was given the 9 landscapes as individual images and asked to choose the one image that best corresponded to the landscape in which he or she grew up. None of our sample populations had any difficulties understanding the task or visually grasping the two-dimensional stimulus. For each respondent the following background variables were collected: gender, age, place of birth and current place of residence.

#### Analyses

The answers from most of the respondents were not consistent in the sense that the pictures could not be uniquely ranked from the 36 preferences from one person. For example, if picture 1 is preferred when it is compared with picture 2 and if picture 2 is preferred when it is compared with picture 3, it is not necessarily true that picture 1 is preferred when it is compared to picture 3.

To model this situation, as well as the factorial design with the pictures described by density of vegetation and topography, we imagine that if a person is shown all the pictures simultaneously, the probability is π_*ij*_, *i* = 1,2,3, *j* = 1,2,3, that the picture with density *i* and topography *j* is chosen as the one to be preferred. According to this assumption the preferences in the pairwise comparisons use the Bradley-Terry model (Bradley and Terry, 1952) where the probability for preferring picture (*i,j*) in favor of picture (*i',j'*) is given by

$$\begin{array}{l} P\left(\prime \prime \text{picture}\;(i,j)\;\;\text{is}\;\text{preferred}\;\text{in}\;\text{favor}\;\text{of}\;\text{picture}\;\left(i^{\prime },j^{\prime }\right)\prime \prime \right)\\ \;=\frac{\pi_{\left(i,j\right)}}{\pi_{\left(i,j\right)}+\pi_{\left(i^{\prime },j^{\prime }\right)}} \end{array}$$

To estimate the parameters in this model, a generalized logit model is used with a picture *R* as reference and a factorial design with density, topography and the interaction between density and topography. Hence

$$\begin{array}{l} \log\frac{\pi_{\left(i,j\right)}}{\pi_{R}}=\mu +\alpha_{i}+\beta_{j}+{\left(\alpha \beta \right)}_{ij} \end{array}$$

Based on this model, a logit model in PROC GENMOD in SAS^2^ can be used since

$$\begin{array}{l} \text{logit}\left(\frac{\pi_{\left(i,j\right)}}{\pi_{\left(i,j\right)}+\pi_{\left(i^{\prime },j^{\prime }\right)}}\right)=\text{log}\left(\frac{\pi_{\left(i,j\right)}/\left(\pi_{\left(i,j\right)}+\pi_{\left(i^{\prime },j^{\prime }\right)}\right)}{1-\pi_{\left(i,j\right)}/\left(\pi_{\left(i,j\right)}+\pi_{\left(i^{\prime },j^{\prime }\right)}\right)}\right)\\ =\text{log}\left(\frac{\pi_{\left(i,j\right)}/\left(\pi_{\left(i,j\right)}+\pi_{\left(i^{\prime },j^{\prime }\right)}\right)}{\pi_{\left(i^{\prime },j^{\prime }\right)}/\left(\pi_{\left(i,j\right)}+\pi_{\left(i^{\prime },j^{\prime }\right)}\right)}\right)=\text{log}\left(\frac{\pi_{\left(i,j\right)}}{\pi_{\left(i^{\prime },j^{\prime }\right)}}\right)\\ =\text{log}\left(\frac{\pi_{\left(i,j\right)}}{\pi_{R}}\right)-\log\left(\frac{\pi_{\left(i^{\prime },j^{\prime }\right)}}{\pi_{R}}\right)\\ =\mu +\alpha_{i}+\beta_{j}+{\left(\alpha \beta \right)}_{ij}-\left(\mu +\alpha_{i}\prime +\beta_{j}\prime +{\left(\alpha \beta \right)}_{i\prime j\prime }\right)\\ =\alpha_{i}-\;\alpha_{i}\prime +\beta_{j}-\;\beta_{j}\prime +{\left(\alpha \beta \right)}_{ij}-{\left(\alpha \beta \right)}_{i\prime j\prime } \end{array}$$

Contrasts were created to test for main effects and interactions in this model (where *p* ≤ 0.05 was considered significant).

Letters were used to illustrate the differences between the pictures for each language group and to avoid the problem of mass significance the adjustment by Holm-Bonferroni was used with the family-wise error rate 5%.

For the comparison between preference and the picture that best corresponded to the landscape where they grew up, we noted for each person whether the picture where they grew up also was the most preferred picture when summarizing the pairwise comparisons. To see if the language groups had different tendencies for preference, the percentage of individuals who preferred the picture where they grew up was calculated.

## Results

**Figure 3:**
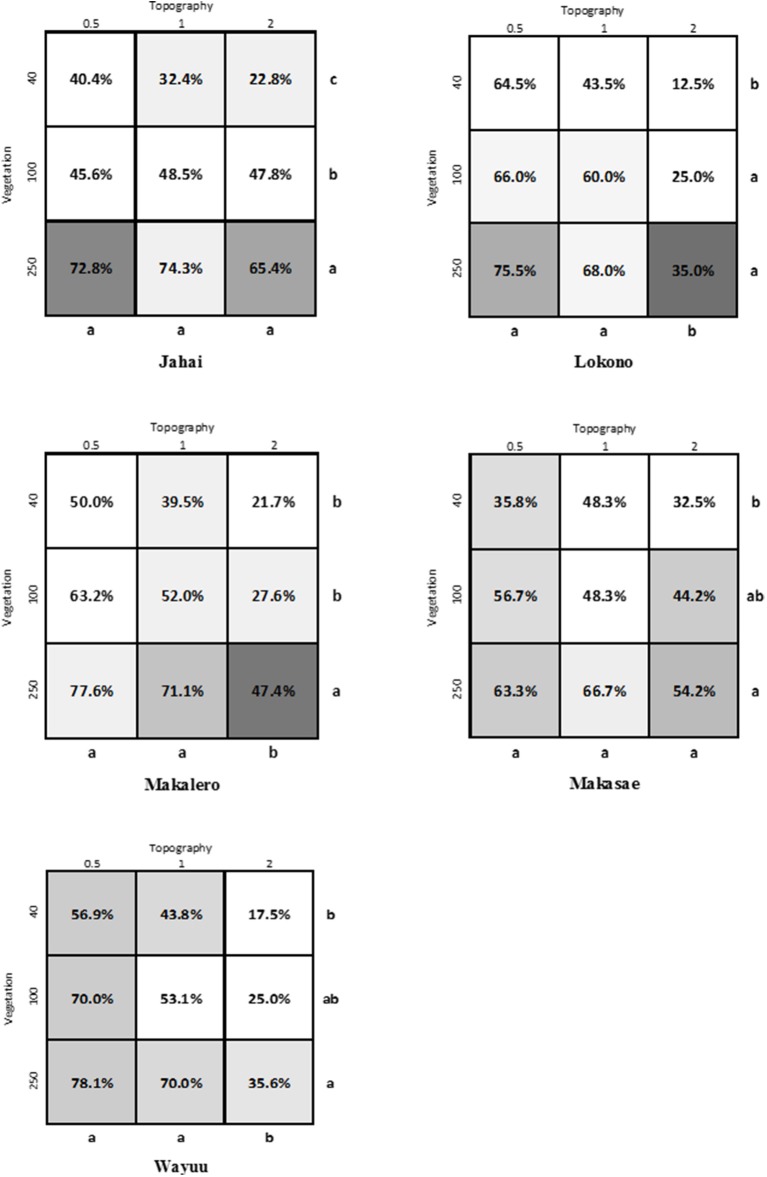
Results for the five non-Western populations. The gray scale indicates whether that image was chosen as an image similar to the landscape in which they grew up; a dark gray indicates that a large part of the participants chose it as the landscape in which they grew up (cf. Table 2). The percentage given is the overall percentage that image was chosen when it was showed. For these populations the contrasts in PROC GENMOD in SAS showed no significant interaction between topography and vegetation and therefore the differences between the levels of the main effects topography and vegetation, respectively, are illustrated by the letters. The same letter (of letters a, b, c next to rows and columns) indicate no significant difference between the levels based on the contrasts. To avoid mass significance, the *p*-values are adjusted with Holm-Bonferroni's method (at *p* ≤ 0.05).

**Figure 4:**
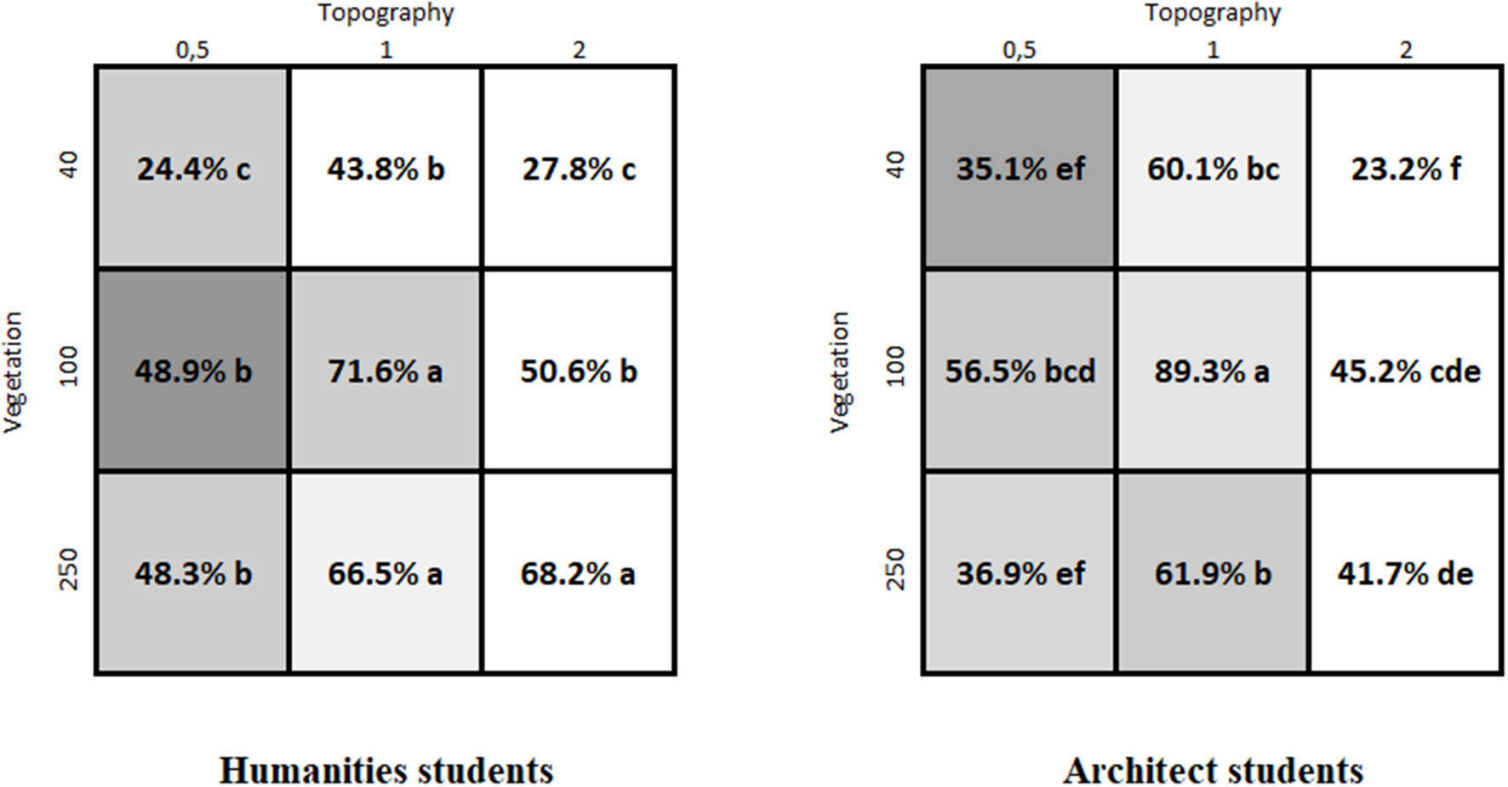
Results for the two Western, Swedish student samples. The gray scale indicates whether that image was chosen as an image similar to the landscape in which they grew up; a dark gray indicates that a large part of the participants chose it as the landscape in which they grew up (cf. Table 2). The percentage given is the overall percentage that image was chosen when it was showed. For these populations the contrasts in PROC GENMOD in SAS showed a significant interaction between topography and vegetation and the letters within the matrix compare different pictures. The same letter (of letters a, b, c, etc. in the matrix) indicate no significant difference between the pictures based on the contrasts. To avoid mass significance, the *p*-values are adjusted with Holm-Bonferroni's method (at *p* ≤ 0.05).

First, looking at the effects of the factors of topography and density of vegetation on preference we found a difference between the non-Western sample groups and the two Western (Swedish) groups. For the five non-Western groups — Jahai, Lokono, Makalero, Makasae and Wayuu — there were no significant interactions between topography and density of vegetation (see Figure 3) and therefore it is sufficient to indicate the differences within the main effects of topography and density. In contrast, for the two Swedish samples there were interaction effects between topography and density of vegetation and the interpretation is more complicated (see Figure 4). With a significant interaction it is not possible to separate the analysis into an analysis of the main effects only and for this reason the letters in the figures show a comparison of all nine pictures simultaneously.

Second, examining which images were most preferred, we again note a difference between the non-Western and the Western (Swedish) groups. For the Jahai, Lokono, Makalero, Makasae, and Wayuu, the general pattern is that the most preferred images are those in the bottom left corner of the matrix (see Figures 1, 3), i.e., flatter landscapes with high density of vegetation. The preferences of the two Swedish samples instead show the highest preference for the image in the center of the matrix, (see Figures 1, 4), i.e., the image with mid-level of topography and mid-level of vegetation density. This preference is particularly pronounced in the sample consisting of students of landscape architecture.

Third, considering effects of familiarity on preferences, we once again note a difference between the sample populations, which fall into three groups (see Table 2). For Lokono, Makasae and Jahai preferences coincide to quite a large degree with the landscape in which they grew up; 50–60% of respondents prefer the image also chosen as most resembling the landscape in which they grew up. This is different from the Swedish respondents, who show little tendency (< 10%) to pick the landscape they grew up in as the preferred one. Makalero and Wayuu make up a third group with a slightly higher tendency, around 15%.

**Table 2:** Preferences for the landscape in which the respondents grew up.

**Grew up**	**1**	**2**	**3**	**4**	**5**	**6**	**7**	**8**	**9**	**Total**	**Percent**
Lokono	6/8	0/1	8/14	–	0/1	1/1	–	–	–	15/25	60.0
Makasae	0/2	2/2	3/3	–	–	0/1	–	1/3	2/4	8/15	53.3
Jahai	–	–	5/8	1/1	–	1/1	0/1	–	2/6	9/17	52.9
Makalero	–	–	1/1	0/1	0/1	0/4	–	0/1	2/9	3/17	17.6
Wayuu	1/4	1/4	0/4	0/3	–	1/3	–	–	0/2	3/20	15.0
SLU	0/7	0/4	0/3	0/1	1/2	1/4	–	–	–	2/21	9.5
LundUniv	1/4	0/9	1/4	–	0/4	0/1	–	–	–	2/22	9.1
Total	8/25	3/20	18/37	1/6	1/8	4/15	0/1	1/4	6/21	42/137	30.7

*The columns show the responses for the nine images respectively and in the column to the far right the percentage of respondents preferring their home picture. Cells without numbers mean that no one in the sample chose this image as resembling the landscape in which they grew up. For example for Lokono; first column image 1 numbers state 6/8, meaning that image 1 was chosen as most resembling the landscape in which I grew up by 8 respondents, and of those 8, 6 also preferred this image*.

## Discussion

The main objective of this study is to test to what degree preferences for natural landscapes differ across human populations, and if a manifestly cross-cultural sample reveals a universal preference for moderate to high openness predicted by theories in the field and previous empirical research. Of particular interest is also the degree to which the responses from the commonly used Western urbanized student-based samples correspond to those of other populations. The study used culturally neutral representations of landscapes which varied only in two gross structural components, topography and density of vegetation. Given the theoretical claims, a high consensus between populations is expected for such stimuli.

The results show that the populations fall into two groups. The preference of the two Western (Swedish) samples, whose preferences were influenced by interactions between topography and vegetation density. On the other hand no such interaction effects were present for the other five populations. For the non-Western, population main effects of topography and vegetation density were found. This indicates that strategies for how judgements are made, at least when using photographically controlled representations of landscape, might differ between Western urbanized schooled respondents and the indigenous populations who still have a close relationship to the land. The Swedish groups displayed interaction effects between topography and vegetation which suggests that the respondents from this group were not separating these variables when they perceived and evaluated the landscape. In contrast, the other groups considered the variables separately in their evaluation and therefore appear to respond more directly to topography and density as separate properties of the landscape.

The result could also reflect that the Swedish student respondents are more affected and tuned to the test situations. This would suggest that they try to understand the parameters of the task and respond accordingly rather than responding and choosing images based on preference.

Furthermore, the pattern of preference for openness differed between the non-Western and the Swedish samples. For the two Swedish samples, their overall highest preference was for the image with medium density and medium variation of topography. This tendency was particularly strong in the students of landscape architecture, who can be considered to be experts trained in landscape evaluation. The students of landscape architecture thus demonstrate the preference for a moderate openness asserted by theory (Appleton, 1975; Orians, 1980; Kaplan and Kaplan, 1989) and previous empirical work (Balling and Falk, 1982; Ulrich, 1993; Tveit, 2009; Falk and Balling, 2010). The five non-Western samples, on the other hand, favored the highest level of vegetation density, i.e., a low degree of openness.

Hence the indigenous samples favored more forested settings. It has been discussed that alternatively to the Savannah Theory humans evolved in closed forested settings and some limited empirical evidence showing preferences for forested biomes have been put forward as support (Han, 2007).

Although both Swedish student samples had the highest preference for the center image of the matrix, i.e., a landscape with medium level of both variables, there was also some difference between the student groups. The landscape architects showed high preference for all the mid topography images, while the humanities students did not select the one image with mid topography and low density of vegetation to the same degree. Another image for which there was a notable difference between the student groups is the landscape with high density of vegetation and high topography, which was preferred by the humanities students but not by the landscape architect students. The humanities students appear to be somewhat more in favor of higher vegetation density than the landscape architect students. Herzog et al. (2000) reported a similar result, with landscape architect students having higher preference for open scene categories than a group called regular students. Also, in a study by Tveit (2009), openness was a predictor of preference for students from landscape oriented fields, but not so for respondents from the general public. However, in the study by Herzog et al. (2000) regular students had the lowest preference for the vegetation category compared to the other student groups (landscape architects and aboriginal students), which is contrary to our finding with humanities students tending to favor more vegetation in the images.

It should be noted that inconsistencies between studies could partly be due to the difference in how the content of the stimuli is controlled and labeled. A factor like openness or vegetation can either be completely controlled with visualizations like in our study, a priori calculated based on coverage in a photograph (Tveit, 2009; Zhao et al., 2013b), based on ratings by judges (Han, 2007) or derived from the respondents' preference ratings (Herzog et al., 2000). High control of stimuli content would be preferable in future research to enable comparisons between studies and replications.

Based on the above possible differences in judgement strategies and in degree of openness favored by the groups one might speculate that the respondents in the two Swedish student groups are also making judgments more guided by the range of stimuli included and tend to opt for the most balanced stimuli in relation to those factors that vary. In other words, we may observe an effect of schooling and familiarity with surveys, which with expert knowledge becomes even more pronounced. The other indigenous population groups generally preferred the lower left hand corner of the matrix, i.e., flatter and more densely vegetated landscapes. The non-significant interaction also shows that the judgements are made more distinctly based on differences in topography and density of vegetation independently.

Familiarity or attachment to the type of landscape where one has grown up is a factor commonly considered as a predictor of landscape preferences. In this study the respondents were asked to choose the one of the nine stimuli that they thought resembled best the place where they grew up, offering us a possibility to test if the topographical variation and vegetation density of their landscape of origin was affecting current preferences. Results again showed that the populations differed in this respect. Some of the non-Western groups (Lokono, Makasae and Jahai) showed quite a strong tendency to prefer their landscape of origin (50–60%, see Table 2), while again the two Swedish samples were clearly different with a low percentage (< 10%) preferring their landscape of origin. This echoes the results of Sonnenfeld (1967), which showed that groups which were free from subsistence concerns had a stronger preference for exotic landscapes. It is possible that the students could be more likely to favor landscapes that are novel and interesting while preferences in some of the indigenous groups, still living in and off the land, could be more guided by experiences of how the environment can be utilized.

### Limitations due to sample populations

An important purpose of this study was to include small-scale indigenous communities. This however is associated with limitations to our ability to control for or test the impact of factors like age, gender, and educational status. Similarly, obtaining comparable conditions concerning rural or urban residence or education level within all samples has not been possible. Rather, in this study the difference in residence and education occur between the Western and non-Western samples, as a result of the diverse conditions in which these samples can be found. These limitations must be taken into account when interpreting the results. Education and urban vs. rural residence would be factors of interest to further elaborate on in future studies since these factors have been suggested to explain differences between groups in cross cultural visual landscape preference studies (Yu, 1995). Concerning the statistical analysis the sample sizes from each group is not large but we can see the differences in patterns of preferences between the groups, to some extent because the model with a factorial design and all pairs of pictures effectively uses the information from the preference study. It is not necessarily true that larger samples should make a much better comparison between the populations because the sampling procedure of persons could not guarantee that the samples are random samples from the populations.

## Conclusion

Our results bear evidence to clear differences between Western student samples and indigenous communities in the way landscapes are evaluated, what landscapes contain, and the impact of previous experiences. The non-Western groups responded more directly to topography and density of vegetation as specific properties of the landscape when evaluating preference. In contrast, the Western student groups, did not separate the properties out to the same extent but showed interaction effects between topography and vegetation. This could be because this sample might evaluate the landscape more as a whole. Alternatively, it might reflect a greater understanding of the type of survey and the parameters tested and a production of responses accordingly.

The study did show that there were internal similarities between the two Western samples on the one hand, and between the five non-Western samples on the other. To some extent this supports the idea of consensus in preference, not universally but within those categories respectively.

Our study shows that strong universal consensus with preferences for moderate to high openness—asserted by current theory mostly on the basis of studies drawing on Western and/or urbanized sample populations—can be contested if the empirical venture is expanded to include diverse and small-scale indigenous communities living in non-urban environments. Thus, claims of innate preferences, driven by evolution, for semi-open savannah-style environments are not supported by our cross-cultural sample. Our results rather point to strong consensus in preference for densely forested environments for the non-Western samples. However, in order for any generalizations to be made in this regard, additional preference studies from an even broader sample of communities in a wider range of ecologies will be necessary.

## Ethics statement

The research program of which this study was part was approved by the Regional Ethical Review Board in Lund and determined by the Board not to fall under the Ethical Review Act due to the non-sensitive and non-invasive nature of the research. All subjects in the literate sample populations gave written informed consent in accordance with the Declaration of Helsinki. All subjects in the non-literate sample populations gave informed consent according to a protocol of oral approval developed in full accordance with the standards and guidelines of the European Commission's Seventh Framework Program as well as the CODEX of the Swedish Research Council, and the recommendations of the Code of Conduct of the Volkswagen Foundation's Documentation of Endangered Languages program and the Code of Ethics of the International Society of Ethnobiology.

## Author contributions

CH co-desinged the survey in collaboration with ÅOS. Took part in the analysis and intepretation of the results and the writing of the paper. ÅOS co-designed the survey in collaboration with CH. Developed the imagery. Took part in the analysis of the results and the writing of the paper. J-EE developed and carried out the statistical analysis of the survey. Contributed to the writing of method and result section. FA, KR, JH, and NB carried out the survey in field and provided guidance for local adaptation of the questions and intepretation of results for local areas. Contributed to the writing of the paper, particularly regarding description of local groups. NB co-ordinated the project and the carrying out of the survey.

### Conflict of interest statement

The authors declare that the research was conducted in the absence of any commercial or financial relationships that could be construed as a potential conflict of interest.

## Footnotes

^1^Henrich et al. (2010) have dubbed such populations “Western, Educated, Industrialized, Rich, Democratic”—or WEIRD.

^2^SAS 9.4. SAS Institute Inc., Cary, NC, USA. All Rights Reserved.
